# F186. TAPETUM ABNORMALITIES IN FIRST-EPISODE PSYCHOSIS AND RELATIONSHIP TO SYMPTOM SEVERITY AND DURATION OF UNTREATED PSYCHOSIS

**DOI:** 10.1093/schbul/sby017.717

**Published:** 2018-04-01

**Authors:** Sun Woo Lee, Arira Lee, Kang-Soo Lee, Jeong Hoon Kim, Sang-Hyuk Lee

**Affiliations:** 1 CHA Bundang Medical Center, CHA University; 2 Bundang Jesaeng Hospital

## Abstract

**Background:**

Diffusion tensor imaging (DTI) studies have shown white-matter (WM) abnormalities in most corpus callosum segments in first-episode psychosis (FEP) patients. However, the tapetum, one of its sub-segments, is not fully studied until now. This study focuses on tapetum and its relationship with psychotic disorders using DTI with symptom severities and duration of untreated psychosis (DUP).

**Methods:**

Ninety-five FEP patients and thirty healthy controls (HCs) were enrolled. Tapetum, region of interest was extracted using 3D slicer and tract-based spatial statistics (TBSS) were used for DTI analysis. All patients were assessed DUP and clinical symptoms by Scale for the Assessment of Positive Symptoms (SAPS), Scale for the Assessment of Negative Symptoms (SANS) at baseline.

**Results:**

TBSS revealed that fractional anisotropy (FA) values of tapetum in FEP is significantly decreased compared to HCs (p<0.01, FWE-corrected). Exploratory correlational analysis revealed significant negative correlations between FA values of left tapetum and baseline SAPS total scores (r =-0.278, p<0.05), bizarre behavior subscale scores (r =-0.310, p<0.01), positive formal thought disorder subscale scores (r =-0.348, p<0.01), inappropriate affect subscale scores (r =-0.315, p<0.01) and SANS avolition-apathy subscale scores (r =-0.257, p<0.05). Also FA values of left tapetum was negatively correlated with DUP (r =-0.426, p<0.00).

**Discussion:**

FEP patients show a significant reduction in WM integrity of left tapetum, also show correlation with clinical symptoms and DUP. These results suggested that left tapetum may play a role in symptom severities and prognosis in FEP.

